# The complete chloroplast genome of *Tulipa sinkiangensis* Z. M. Mao (Liliaceae) with multi-flower

**DOI:** 10.1080/23802359.2022.2160217

**Published:** 2023-01-02

**Authors:** Guimei Xing, Huihua Zhang, Yanqiu Zhang, Jiaojiao Lu, Tianyu Wu, Zengzhi Tian, Lianwei Qu

**Affiliations:** a Institute of Floriculture, Liaoning Academy of Agricultural Sciences, Shenyang, China; b Liaoning Provincial Key Laboratory of Floriculture, Shenyang, China

**Keywords:** *Tulipa* L., chloroplast genome, evolution

## Abstract

*Tulipa sinkiangensis* Z. M. Mao 1980 is endemic to Xinjiang Province, China. In this study, we reported the complete chloroplast genome of *T. sinkiangensis*. The complete chloroplast genome of *T. sinkiangensis* comprises 151,929 bp and was divided into four typical regions: a large single-copy region of 82,062 bp, a pair of inverse repeats of 26,361 bp each, and a small single-copy region of 17,145 bp. A total of 136 genes were identified in this chloroplast, of which 87 were protein-coding, 38 were tRNA, eight were rRNA, and three were pseudogene. The results of this study will provide valuable information for understanding evolution and identification of different species belonging to genus *Tulipa*.

*Tulipa* L. is a genus of perennial herbaceous plant in the Liliaceae family. The number of species in this genus ranges from about 45 (Stork 1984) to more than 130 (Diana 2013). The Pamir Alai and Tianshan Mountains are primary gene resource centers for plants belonging to genus *Tulipa* (Hoog 1973). More than 10% *Tulipa* species were reported as being native to China (Tang and Wang 1980; Han 2014). Germplasm resources have been investigated in China (Jiao et al. 2015). Such studies have concentrated on describing its morphological variation (Chen et al. 2001; Mei and Tan 2006; Jiao et al. 2015). In recent years, the chloroplast genome has been used to study the genetic diversity and phylogenetic relationship in *Tulipa* species (Zhou et al. 2019; Ju, Shi, et al. 2020; Ju, Tang, et al. 2020). In this study, we worked on *T. sinkiangensis* Z. M. Mao 1980 which is a endemic species in China. The aim is to provide the complete chloroplast genome sequence of *T. sinkiangensis*. This is a necessary process to comprehend its phylogenetic relationships with other *Tulipa* species. Samples of *T. sinkiangensis* used in this study were collected from the low mountain slopes around Urumqi city (87°34′E, 43°47′ N, 924 m altitude, Xinjiang Province, China). The corresponding voucher specimen (no. 15006) was deposited in the Liaoning Academy of Agricultural Sciences (https://www.laas.cn/; the contact person is Huihua Zhang, 731932759@qq.com).

The total DNA of *T. sinkiangensis* was extracted from its leaves using the modified CTAB method (Doyle 1987; Yang et al. 2014). Sequencing libraries were generated using a TruSeq DNA Sample Preparation Kit (Illumina, San Diego, CA). Genome sequencing was performed using Illumina NovaSeq platform. The filtered reads were assembled by SPAdes v3.10.1 (Bankevich et al. 2012) and the assembled genome was annotated by an integrated plastome sequence annotator CPGAVAS2 (Shi et al. 2019). The chloroplast genome sequence of *T. sinkiangensis* was submitted to the NCBI database with the accession number OL350837. Complete chloroplast genome had a typical quadripartite structure, which was 151,929 bp in length with GC content of 36.69%. It contained a large single-copy region (LSC) of 82,062 bp, a small single-copy region (SSC) of 17,145 bp, and two inverted repeat (IR) regions of 26,361 bp. A total of 136 genes were successfully annotated, including 87 protein-codon genes, 38 tRNA genes, eight rRNA genes, and three pseudogenes. To understand the phylogenetic position of *T. sinkiangensis* with its close relatives, phylogenetic analysis was performed based on 24 complete chloroplast genomes of various species in Liliaceae. These genomes were aligned by MAFFT v7.427 (Katoh and Standley 2013). Evolution history was inferred by using maximum-likelihood method based on Tamura–Nei model in RAxML v8.2.10. Bootstrap value was calculated based on 1000 replicate analysis. Results showed that *T. sinkiangensis*, *T. buhseana*, *T. altaica*, and *T. iliensis* were clustered in a unique clade in the *Tulipa* genus (Figure 1).

**Figure 1.**
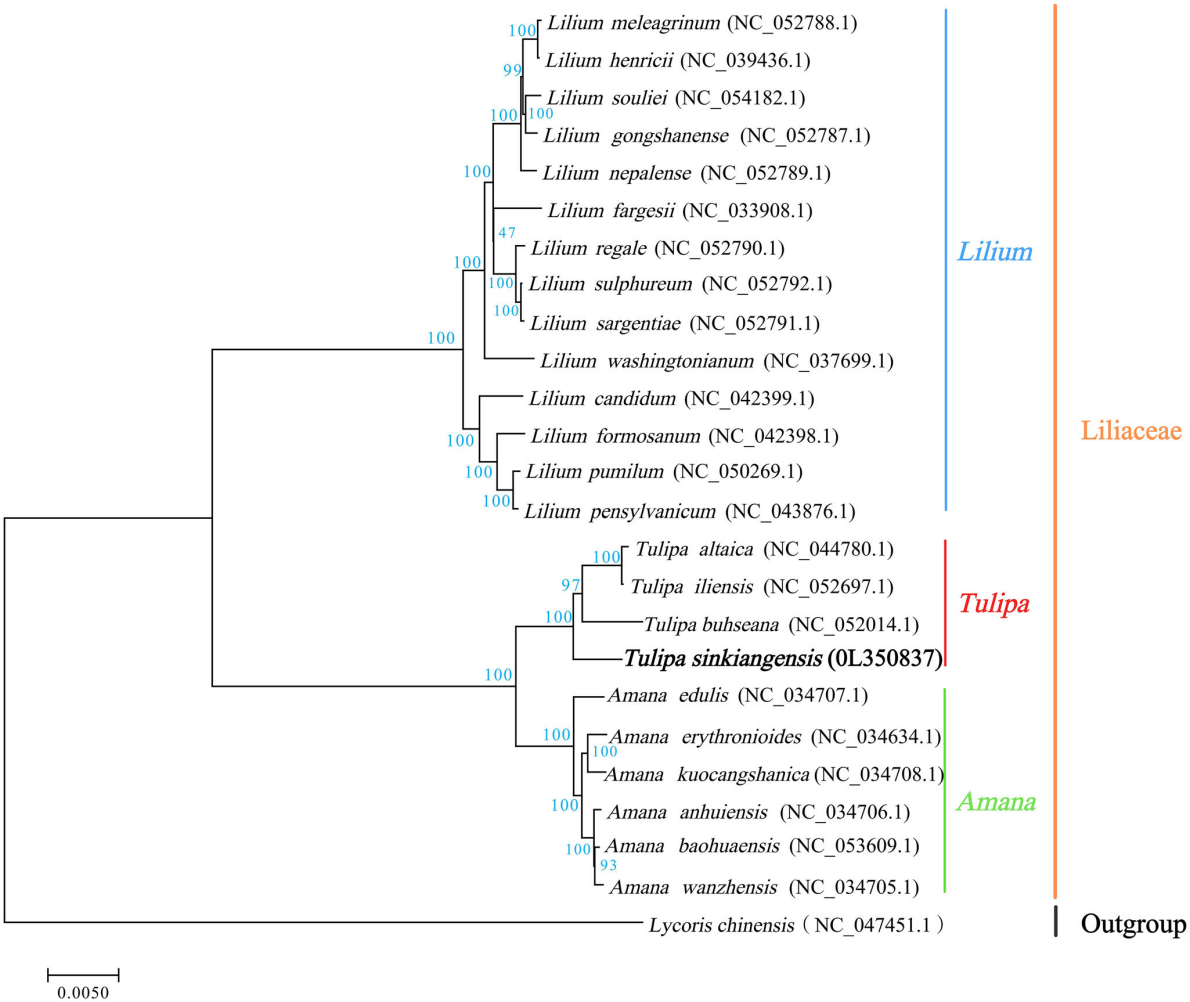
The maximum-likelihood phylogenetic tree was reconstructed based on the complete chloroplast genome sequences of 24 species in Liliaceae with *Lycoris chinensis* (NC_047451.1) as the outgroup. Bootstrap values based on 1000 replicates.

## Data Availability

The genome sequence data that support the findings of this study are openly available in GenBank of NCBI at (https://www.ncbi.nlm.nih.gov/) under the accession No. OL350837. The associated BioProject, SRA, and Bio-Sample numbers are PRJNA834607, SRR19070076, and SAMN28052728, respectively.
